# Identification of the Augmin Complex in the Filamentous Fungus *Aspergillus nidulans*

**DOI:** 10.1371/journal.pone.0101471

**Published:** 2014-07-08

**Authors:** Tomoya Edzuka, Lixy Yamada, Kyoko Kanamaru, Hitoshi Sawada, Gohta Goshima

**Affiliations:** 1 Division of Biological Science, Graduate School of Science, Nagoya University, Furo-cho, Chikusa-ku, Nagoya, Japan; 2 Sugashima Marine Biological Laboratory, Graduate School of Science, Nagoya University, Sugashima, Toba, Japan; 3 Department of Biological Mechanisms and Functions, Graduate School of Bioagricultural Sciences, Nagoya University, Furo-cho, Chikusa-ku, Nagoya, Japan; Cancer Research UK London Research Institute, United Kingdom

## Abstract

Augmin is a protein complex that binds to spindle microtubules (MTs), recruits the potent MT nucleator, γ-tubulin, and thereby promotes the centrosome-independent MT generation within mitotic and meiotic spindles. Augmin is essential for acentrosomal spindle assembly, which is commonly observed during mitosis in plants and meiosis in female animals. In many animal somatic cells that possess centrosomes, the centrosome- and augmin-dependent mechanisms work cooperatively for efficient spindle assembly and cytokinesis. Yeasts have lost the augmin genes during evolution. It is hypothesized that their robust MT nucleation from the spindle pole body (SPB), the centrosome-equivalent structure in fungi, compensates for the lack of augmin. Intriguingly, however, a gene homologous to an augmin subunit (Aug6/AUGF) has been found in the genome of filamentous fungi, which has the SPB as a robust MT nucleation centre. Here, we aimed to clarify if the augmin complex is present in filamentous fungi and to identify its role in mitosis. By analysing the Aug6-like gene in the filamentous fungus *Aspergillus nidulans*, we found that it forms a large complex with several other proteins that share weak but significant homology to known augmin subunits. In *A. nidulans*, augmin was enriched at the SPB and also associated with spindle MTs during mitosis. However, the augmin gene disruptants did not exhibit growth defects under normal, checkpoint-deficient, or MT-destabilised conditions. Moreover, we obtained no evidence that *A. nidulans* augmin plays a role in γ-tubulin recruitment or in mitotic cell division. Our study uncovered the conservation of the augmin complex in the fungal species, and further suggests that augmin has several functions, besides mitotic spindle MT nucleation, that are yet to be identified.

## Introduction

Formation of microtubules (MTs) is essential for various cellular events such as mitosis, organelle transport, and cell growth. MTs are nucleated at multiple locations. In animal somatic cells, the dominant MT nucleation site is the centrosome, at which the potent MT nucleator, the γ-tubulin complex (γ-TuC), accumulates and serves as the seed for new MT formation [1]. In some systems such as *Drosophila* or fungi, γ-TuC at the centrosome can be present in 2 forms: a smaller complex γ-TuSC consisting of γ-tubulin and GCP2/3 subunits, and a larger ring complex of γ-TuRC that additionally contains GCP4–6 and NEDD1 subunits [2]–[4]. Cells also possess acentrosomal MT nucleation mechanisms, such as nucleation at the Golgi apparatus, which is observed in interphase, or at the chromatin-proximal region during mitosis [5], [6]. MT-dependent MT nucleation is another universal mechanism, in which a new MT is generated at the lattice of existing MTs; in this ‘branching’-type nucleation, γ-TuRC localised to the MT lattice plays a critical role [7]. Augmin is an 8-subunit protein complex required for the MT-dependent MT nucleation during mitosis [8], [9]. In the absence of augmin, γ-tubulin localisation on the spindle MTs, but not at the centrosome, is specifically reduced, such that intra-spindle MT nucleation is impaired (centrosomal nucleation is not perturbed). Consistent with this phenotype, augmin is localised uniformly to the spindle MTs. On the other hand, the interphase role of augmin has not yet been identified, although it is enriched at the centrosome.

Several studies have shown that augmin is particularly important for cell division in cells that lack centrosomes. In *Drosophila* S2 cells, depletion of augmin was found to have an effect on mitotic spindle formation or chromosome segregation. However, the defect became more pronounced when centrosomal nucleation was co-inhibited by using RNAi against centrosomin, a γ-tubulin recruiter/activator at the centrosome; cells could no longer form a bipolar spindle or properly segregate chromosomes [8]. Furthermore, flies that are null mutants of augmin subunits are viable, indicating that somatic cell division is not completely perturbed [10], [11]. However, they showed a severe defect in meiotic cell division in oocytes, during which the spindle is assembled in the absence of centrosomes. Land plants lack centrosomes, and the depletion of augmin leads to premature disappearance of the phragmoplast, a MT-based bipolar structure required for cytokinesis, due to defects in MT generation during cytokinesis [12], [13]. On the other hand, surveys of the genome sequences of *Caenorhabditis elegans* and yeast have failed to identify genes homologous to any augmin subunits (Aug1–Aug8). Conceivably, these species have robust MT nucleation activity associated with the centrosome or spindle pole body (SPB), and the augmin-mediated mechanism is dispensable for preparing MTs for spindle formation, chromosome segregation, and cytokinesis. In fact, electron microscopy of the spindle of yeast or the filamentous fungus *Ashbya gossypii* indicated that all the spindle MTs originate from the SPB region [14], [15].

**Figure 1:**
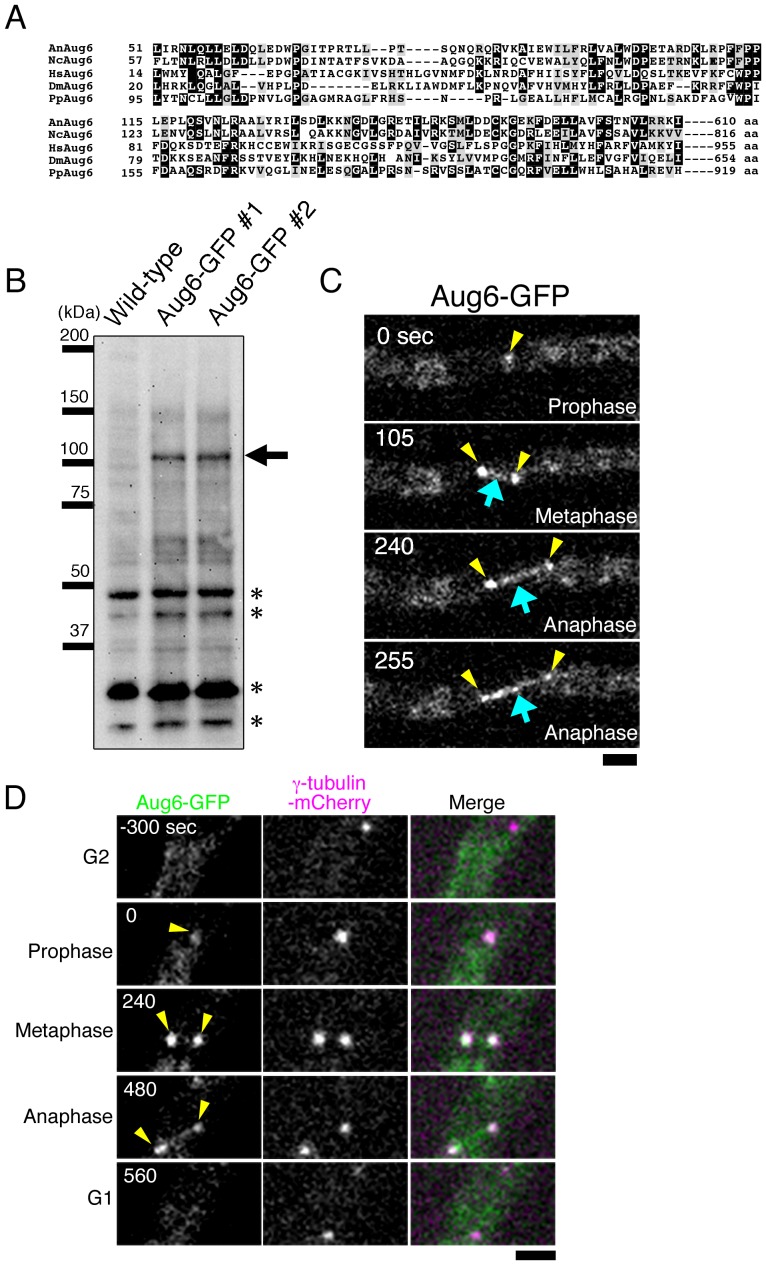
Aug6 is localised at the SPB and the spindle. (A) Sequence alignment of Aug6 proteins from *A. nidulans* (An), the red bread mould *Neurospora crassa* (Nc), *Homo sapiens* (Hs; also called hDgt6/FAM29A/HAUS6), *Drosophila melanogaster* (Dm; also called Dgt6), and the moss *Physcomitrella patens* (Pp). Identical amino acids are boxed, and similar ones are hatched. (B) Detection of Aug6-GFP by immunoblotting. A unique band with the expected molecular weight (arrow) was detected by immunoblotting with the anti-GFP antibody in 2 independent GFP-integrated strains (#1 and #2). Asterisks indicate cross-reactions of the antibody with other proteins. The #2 strain was used throughout this study. (C) Time-lapse imaging of Aug6-GFP during mitosis. Images were acquired every 15 s in a single focal plane. Strong signals were detected at the pole of the spindle (yellow), whereas weak punctate signals were observed along the spindle MT (blue). (D) Time-lapse imaging of Aug6-GFP and γ-tubulin-mCherry. They were co-localised at the SPB during mitosis. See also Movie S1. Bars, 2 µm.

Interestingly, a BLAST homology search has identified an Aug6-like gene in the genome of various non-yeast fungal species, including many species of the filamentous fungi, in which spindle assembly is generally believed to be exclusively mediated by SPB-originated MTs, similar to that in yeast [16] (Fig. 1A). A possible explanation is that this gene is a non-functional remnant of the augmin complex that has been lost during fungal evolution. Alternatively, filamentous fungi may possess the complete augmin complex whose other subunits are highly diverged in their amino acid sequences and are thus difficult to identify using conventional database searches.

In this study, we began with the characterisation of the Aug6-like gene in *A. nidulans*, a popularly used fungal species for cell division research [17], [18]. We found that Aug6 is localised to the mitotic SPB and constitutes a large protein complex that can bind to MTs. Aug6 is associated with several proteins, some of which have weak but significant homology to other animal/plant augmin subunits. Each subunit was co-localised with Aug6. Thus, the augmin complex was present in *A. nidulans*. However, we have obtained no evidence that augmin recruits γ-tubulin or mediates MT-dependent MT nucleation in the spindle. Fungal augmin might have uniquely evolved and possibly plays a yet unknown role.

## Results

### *A. nidulans* Aug6 is localised to the SPB and spindle MTs during mitosis

We sought to determine the intracellular location of the Aug6-like protein (AUGF/AN6187) in *A. nidulans*. To this end, we tagged GFP in frame to the C-terminus of the endogenous *AN6187* gene, followed by time-lapse microscopy (Fig. 1B, C). GFP signals were not detected at specific sites during interphase. However, we observed 1−2 strong punctate signals and weak signals between them for approximately 6 min, reminiscent of the dynamics of mitotic SPB and spindle [19], [20]. We then tagged mCherry to endogenous γ-tubulin, which is known to be localised at the SPB, and imaged GFP and mCherry in living cells (Fig. 1D and Movie S1). As expected, we observed punctate signals of γ-tubulin throughout the cell cycle (we interpret the occasional loss of signal is due to the cell or SPB moving out of the plane of focus). Upon entry into mitosis, Aug6-GFP signals appeared and were closely localised to γ-tubulin-mCherry until the completion of mitosis. We conclude that Aug6 is enriched at the SPB and is also weakly associated with spindle MTs during mitosis.

### Identification of the augmin complex of *A. nidulans*

**Figure 2:**
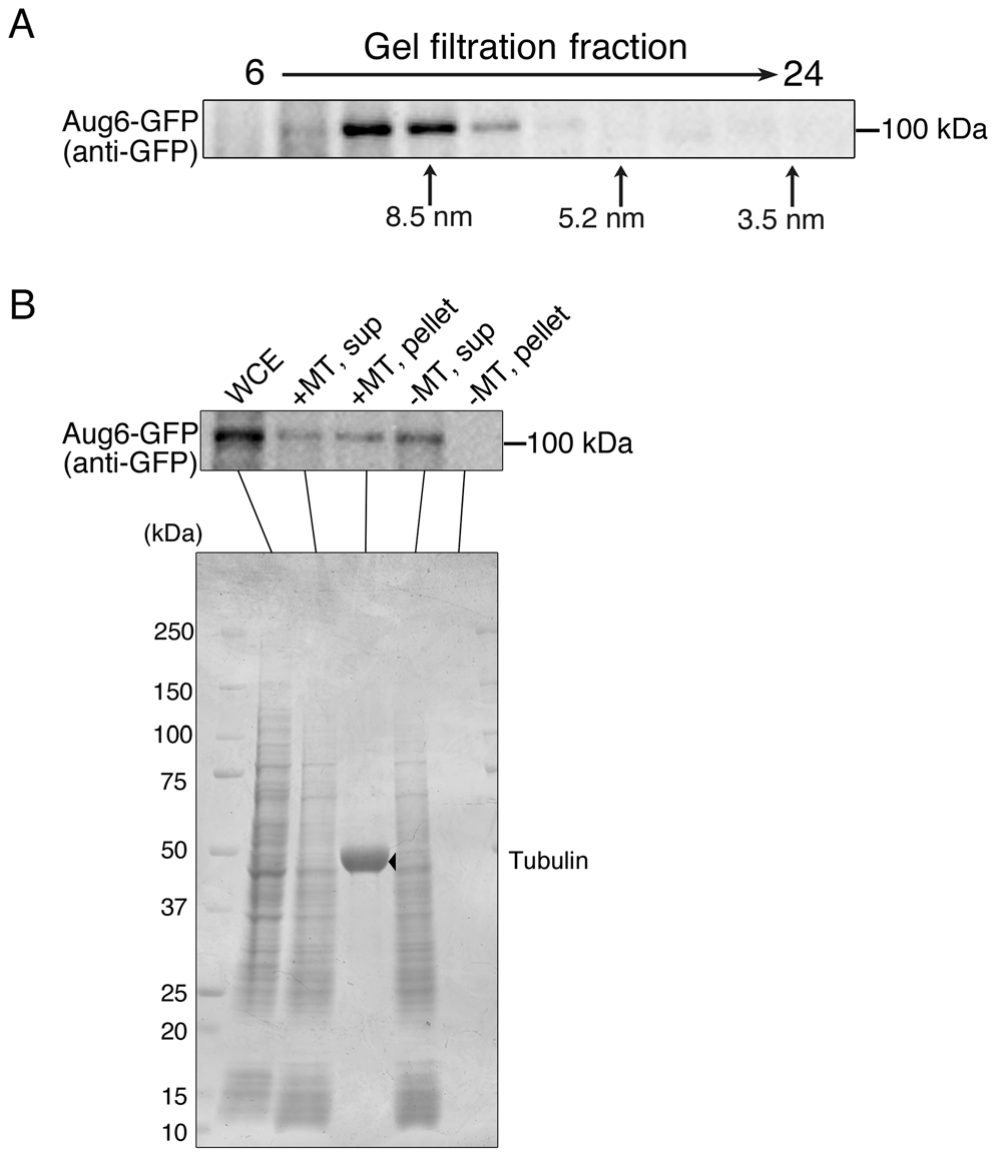
Aug6 forms a large complex and associates with MTs. (A) Gel filtration chromatography followed by immunoblotting of Aug6-GFP. Stokes radiuses estimated by the size markers are shown at the bottom. (B) MT co-sedimentation assay using Aug6-GFP cell extracts and taxol-stabilised MTs. Results of anti-GFP immunoblotting (Aug6) and Coomassie staining (tubulin [arrowhead] and the whole *A. nidulans* proteins) are shown. WCE; whole cell extracts, sup; supernatant after centrifugation, pellet; precipitant after centrifugation.

To test whether the augmin complex containing Aug6 is present in *A. nidulans*, we performed gel filtration chromatography using the Aug6-GFP strain. Interestingly, anti-GFP immunoblotting indicated that Aug6 is a member of a large complex of ≥8.5 nm Stokes radius, reminiscent of the animal or plant augmin (Fig. 2A) [8], [9], [12]. Next, we performed the MT co-sedimentation assay using taxol-stabilised MTs and the whole cell extract of the Aug6-GFP strain (Fig. 2B). Immunoblotting of Aug6-GFP showed that it is co-precipitated with MTs, suggesting that the Aug6-containing complex directly or indirectly binds to MTs. This behaviour is also identical to that of fly Aug6 protein [8].

**Figure 3:**
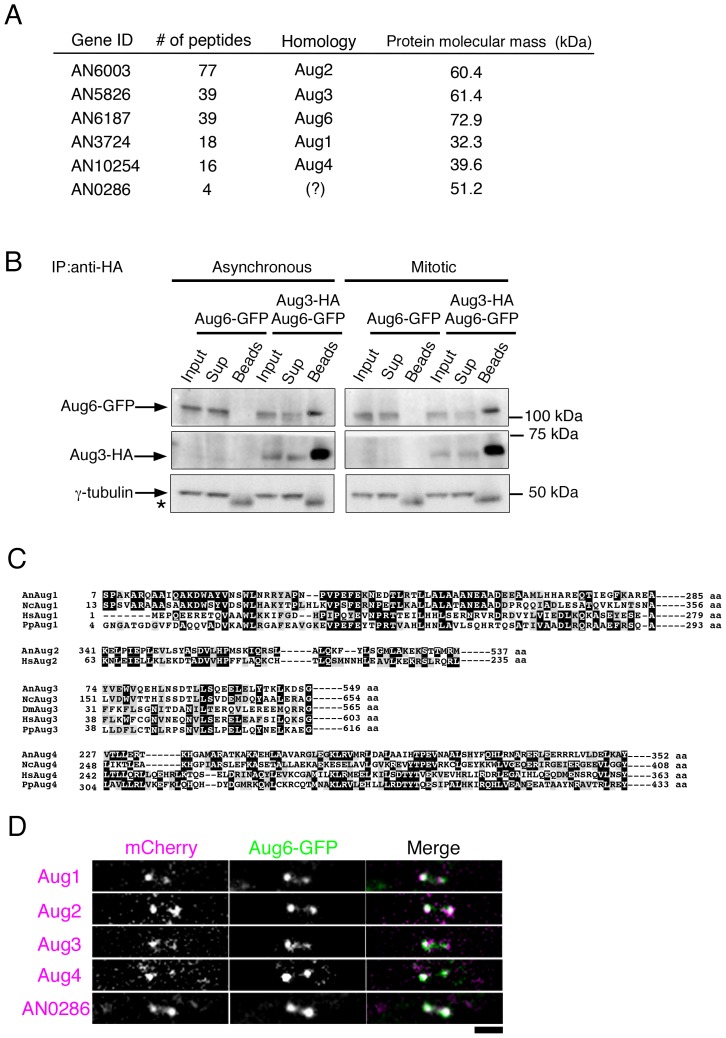
Identification of other augmin subunits that co-precipitate with Aug6. (A) List of putative augmin subunit proteins co-precipitated with Aug6-GFP. The numbers of peptides identified in the Aug6-GFP immunoprecipitants are shown. No homology to known augmin subunits was found for AN0286. (B) Co-precipitation of Aug6-GFP, but not γ-tubulin, with Aug3-HA. Asynchronous and metaphase-accumulated cell cultures were used. Aug3-HA was immunoprecipitated by the anti-HA antibody, followed by immunoblotting of Aug6-GFP or γ-tubulin. Asterisk indicates cross-reaction of the antibody with the IgG proteins. Sup; proteins unbound to the anti-HA beads, Beads; immunoprecipitants. Equal amounts of Input and Sup were loaded, whereas the immunoprecipitants were 19- (Aug6-GFP, Aug3-HA) or 150 (γ-tubulin)-fold concentrated. (C) Amino acid sequence alignment of Aug1, Aug2, Aug3, and Aug4. Sequences from the red bread mould *Neurospora crassa* (Nc), *Drosophila melanogaster* (Dm), *Homo sapiens* (Hs), and the moss *Physcomitrella patens* (Pp) are aligned. (D) Co-localisation of Aug1–4 and AN0286 with Aug6 in metaphase. mCherry-tagged Aug1, Aug2, Aug3, Aug4, or AN0286 (purple) was co-imaged with Aug6-GFP (green) using a spinning-disc confocal microscope. See also Movie S2. Bar, 2 µm.

We next immunoprecipitated Aug6-GFP and identified co-precipitated proteins using mass spectrometry. The PSI-BLAST homology search indicated that many of the identified proteins were orthologues of well-characterised genes unrelated to augmin, such as ribosomal proteins, but they were not reproducibly identified. We could not identify γ-tubulin or other γ-TuRC subunits in the Aug6-precipitated sample. However, we found 4 proteins that shared weak homology to known augmin subunits (Aug1–4); these genes are conserved in many other fungal species, but as of yet have not been identified in yeast (Fig. 3A, C). To investigate intracellular localisation during mitosis, we tagged mCherry to the C-termini of these 4 genes and to the other 9 candidate genes that had no obvious homology to other characterised genes and for which ≥3 peptides were specifically identified by mass spectrometry. Time-lapse imaging showed that the Aug1-, Aug2-, Aug3-, and Aug4-like proteins were co-localised with Aug6-GFP, indicating that these are *bona fide* augmin subunits (named AUGA, AUGB, AUGC, and AUGD; Fig. 3D, Movie S2).

To confirm that Aug3 and Aug6 are co-precipitated, a reciprocal immunoprecipitation was performed using asynchronous culture; Aug3-HA was immunoprecipitated with the anti-HA antibody, followed by mass spectrometry or immunoblotting. In this experiment, Aug6 was co-precipitated with Aug3-HA, whereas γ-tubulin was not (Fig. 3B, left, shows immunoblotting. Mass spectrometry identified 76 peptides of the Aug6 protein, but none of γ-tubulin). No co-precipitation of γ-tubulin was detected even when mitotic cells were accumulated for immunoprecipitation (Fig. 3B, right).

In the mass spectrometry and localisation analyses, we identified another protein (AN0286) that co-localises with Aug6-GFP at the mitotic SPB but does not show significant homology to known augmin subunits in other species (Fig. 3A, D). This gene is conserved in many other fungal species, but not in yeast, animals, or plants. AN0286 was also identified through immunoprecipitation of Aug3-HA fusion protein followed by mass spectrometry, suggesting that this is also an augmin subunit; we speculate that the amino acid sequence has diverged at a point in evolution and the BLAST-based search failed to identify the similarity (see Discussion).

From these results, we conclude that *A. nidulans* has the augmin complex consisting of at least 5 conserved subunits.

### Augmin is not essential for the viability of *A. nidulans*

**Figure 4:**
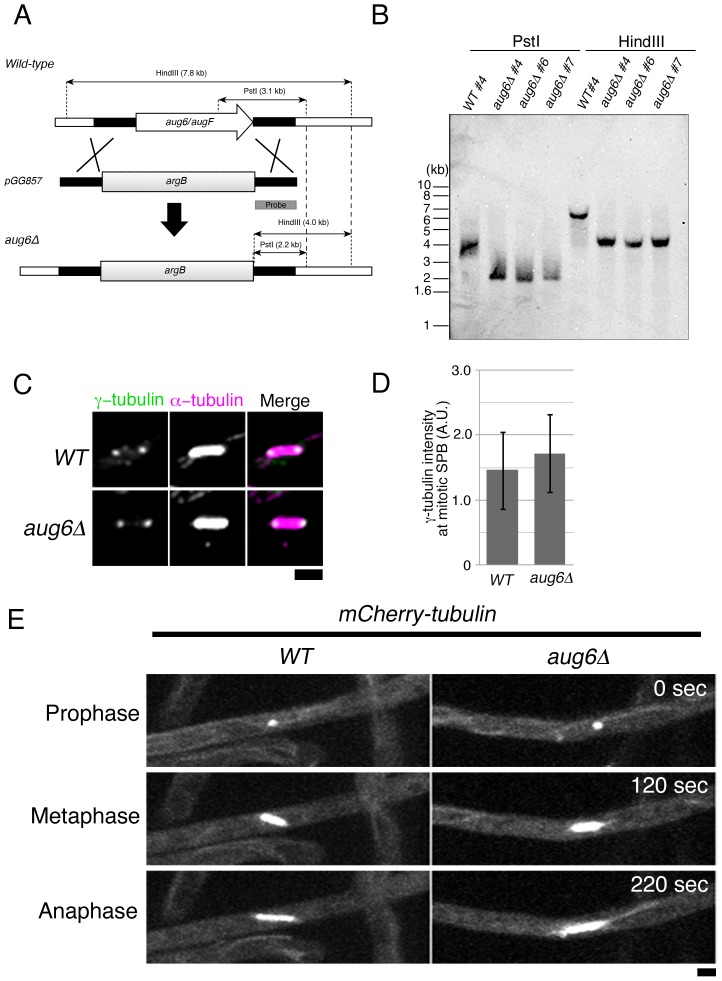
Normal γ-tubulin localisation and mitotic progression in the absence of augmin. (A) Scheme showing the generation of the *aug6* disruptant by one-step gene replacement. (B) Southern blot hybridisation confirmed the *aug6* gene deletion via homologous recombination (3 independently selected strains were analysed). The probe is described in (A). We used the #4 strain in this study. (C, D) γ-tubulin localisation to the SPB was not attenuated in the absence of *aug6*. γ-tubulin signal intensities were quantified in the spindle of WT and *aug6Δ* in 2 independent experiments, and the result from one experiment is displayed (n = 9 hyphae, total 77 and 73 spindles, respectively). Error bars indicate SD. When multiple spindles were analysed in a hypha, the mean value was used as the γ-tubulin signal intensity of the hypha. Maximum projection images of 7 z-sections are displayed. (E) Normal spindle formation and mitotic progression in *aug6Δ*, as monitored by time-lapse imaging of mCherry-tubulin. 25 spindles of 10 WT cells and 22 spindles of 12 *aug6Δ* cells were analysed. Maximum projection images of 5 z-sections are displayed. See also Movie S3. Bar, 2 µm.

**Figure 5:**
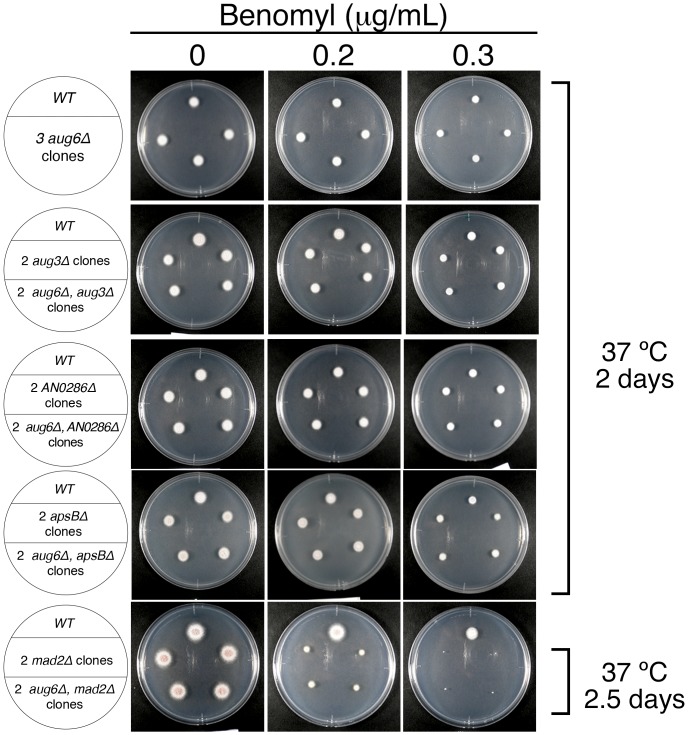
Normal cell proliferation in the absence of augmin. 1×10^4^ conidia were spotted on the medium plate containing 0–0.3 µg/mL benomyl, a MT destabilising drug, and cultured for 2 or 2.5 days at 37°C. Deletion of 1 or 2 augmin subunits did not affect cell proliferation in the WT, *mad2Δ*, or *apsBΔ* background.

To investigate the function of the augmin complex in *A. nidulans*, we deleted the *aug6* gene by using homologous recombination (Fig. 4A, B). The *aug3* and *AN0286* genes were deleted in a similar manner. The gene disruptants were viable and showed no growth defect in the normal culture media (see Fig. 5). Furthermore, conidia (asexual spores) were normally obtained. We conclude that augmin is not essential for the viability of *A. nidulans*.

### Augmin is dispensable for γ-tubulin localisation and spindle MT formation in *A. nidulans* hyphae mitosis

Augmin was identified as a γ-tubulin-localisation factor, and this feature has been conserved in every model system previously investigated [8], [9], [12], [13], [21]. We investigated if γ-tubulin localisation is impaired in the absence of augmin in *A. nidulans* hyphae. To this end, we stained γ-tubulin in control and *aug6Δ* strains. Consistent with previous studies [19], γ-tubulin was specifically localised to the SPB in control cells; spindle localisation was not observed, unlike the findings for animal or plant spindles (Fig. 4C). Surprisingly, we found no difference in γ-tubulin localisation or abundance between control and *aug6Δ* cells (Fig. 4C, D). Time-lapse imaging of mCherry-tubulin in the absence of *aug6* confirmed the formation of the normal-sized bipolar spindle and normal mitotic progression (Fig. 4E, Movie S3; duration of mitosis was 429±13 in wild type [mean ± SEM, n = 10 hyphae] and 426±11 s in *aug6Δ* [n = 12 hyphae]). We conclude that augmin is dispensable for γ-tubulin localisation to the SPB or spindle MT formation in the hyphae of *A. nidulans*.

In *A. nidulans*, 3 outer subunits of the γ-TuRC (GCP4/GCPD, GCP5/GCPE, and GCP6/GCPF) are also dispensable for γ-tubulin localisation to the SPB [3]. However, GCP4, GCP5, and GCP6 play a supportive role in mitotic cell division, and the double disruption with the spindle assembly checkpoint (SAC) component Mad2 (Md2A) showed synthetic growth retardation [3]. We therefore similarly generated the double *aug*/*mad2* disruptant. Surprisingly, we found no growth defect even in this SAC-deficient condition (Fig. 5, bottom left). In *Drosophila* cells, augmin showed a synthetic mitotic defect with centrosomin (Cnn), a centrosome component required for γ-tubulin-mediated nucleation [8]. We tested if this synthetic relationship holds true in *A. nidulans*. We deleted the *apsB* gene, which is homologous to *Drosophila* Cnn and plays an important role in cytoplasmic MT nucleation [22]. However, the double *aug6/apsB* disruptant did not further exaggerate the phenotype (Fig. 5). We speculated that if augmin is involved in spindle function, the augmin disruptant would show a growth alteration in the presence of MT-destabilising drug. We cultured a control strain and various single or double mutants involving augmin genes in the presence of 0.2 or 0.3 µg/mL benomyl, a MT-destabilising drug. However, sensitivity to this compound was identical between control and augmin-deficient strains (Fig. 5). All of these data suggest that augmin plays little or, possibly, no role in mitotic cell division in *A. nidulans*.

## Discussion

This study began with 2 questions: Is the augmin complex present in fungal species that have potent SPB-dependent MT nucleation activity? If it is, then what is the mitotic function of fungal augmin? Our study showed that the large augmin complex is present in the filamentous fungus *A. nidulans*; nevertheless, its deletion does not give rise to any signs of augmin contribution to mitotic cell division.

### Fungal augmin

The presence of Aug6-like proteins in the fungal genome has been reported in a previous study [16]. Our study showed that the Aug6-like protein of *A. nidulans* constitutes a large protein complex and is associated with at least 4 other conserved augmin subunits (Aug1, 2, 3, and 4). The genes homologous to these 4 are found in other fungal species (but not in yeast). We have identified another uncharacterised protein (AN0286) as being a likely augmin subunit, as it was co-precipitated with Aug3 or Aug6 and co-localised with Aug6 at the SPB. It should be noted that the amino acid sequences of the augmin subunits are highly divergent. Even between 2 metazoan species, flies and humans, no clear sequence homologies have been identified for a few subunits [9]. It is therefore possible that additional subunits are also present in the *A. nidulans* genome, which we failed to identify via the BLAST search. Despite the lack of significant amino acid identity, structural similarities have been proposed between fly and human augmin subunits [23]. Similar analysis might be needed to identify other augmin subunits of *A. nidulans*. However, the possibility cannot be excluded that fungal augmin consists of fewer number of subunits.

### Augmin in the mitosis of *A. nidulans*

The reported phenotypes of augmin in every animal and plant species are related to the mitotic cell division process, such as spindle formation or cytokinesis. *A. nidulans* augmin was also found to be enriched at the SPB and spindle during mitosis. However, in the current study, we could not identify these reported phenotypes. A conserved function of augmin in animal and plant cells is MT amplification via γ-TuRC recruitment [8], [9], [12], [13], [21]. However, the complete deletion of *aug6* and/or *aug3* genes did not cause these defects in *A. nidulans* hyphae, even under sensitised conditions such as in the presence of a MT-destabilising drug or absence of Mad2 (*md2A*) or Cnn (*apsB*). This is in contrast to the case of outer subunit genes of the γ-TuRC; depletion of these γ-TuRC-specific subunits (GCP4–6) showed a synthetic growth phenotype with Mad2 [3]. These data reinforce the idea that augmin plays little role in mitosis in *A. nidulans*. Interestingly, a structure-function analysis of augmin and γ-TuC in human cells suggested that the NEDD1 component of γ-TuRC is responsible for augmin association [9], [24] but *A. nidulans* does not seem to possess this particular subunit of γ-TuRC [3]. Fungal augmin and γ-TuRC might not be physically or functionally connected.

Do these data imply that augmin plays no role in *A. nidulans* cells? We think this possibility is less likely. For example, in *Drosophila*, prominent phenotypes were identified in the meiotic spindle in augmin mutants [10]. It would be of interest to investigate if *A. nidulans* augmin plays a role in meiotic spindle formation and chromosome segregation. As another example, a chemical genomics analysis in budding yeast *S. cerevisiae* showed that nearly all genes (≥97%) are important for optimal growth in certain growth conditions [25]. There might be unidentified, redundant machinery for mitotic spindle MT generation in *A. nidulans*. Alternatively, *A. nidulans* augmin might play a yet unidentified role, which was not detected in our assays using normal or benomyl-containing culture media. In this regard, it is noteworthy that although augmin is enriched at the centrosome during the interphase of human cells [9], [16], the function of centrosomal augmin is unknown. The use of *A. nidulans* might have the advantage of identifying the unknown function of augmin, as null strains are available. For example, by identifying the condition under which the augmin disruptant specifically shows growth retardation (e.g. in the presence of a certain chemical compound), a clue to the unidentified augmin function in *A. nidulans* might be obtained. This approach would be difficult using RNAi-based studies in animal or plant cells.

In summary, this study performed using the filamentous fungus, *A. nidulans*, has uncovered the evolutional conservation of the augmin complex. It also implies functional versatility of the augmin complex and/or presence of the unidentified machinery redundant with augmin. Furthermore, our study presents a new and simple genetically/biochemically tractable model for studying functions or molecular structure of the augmin complex.

## Materials and Methods

### Plasmids

The plasmids and PCR primers used in this study are listed in Tables 1 and 2, respectively.

**Table 1:** Plasmids used in this study.

Name	Insert	Marker	Target locus
pGG857	*aug6Δ* construct	*argB*	*aug6*
pGG858	Aug6-Cterm-GFP	*argB*	*aug6*
pGG879	γ-tubulin-Cterm-mCherry	*pyroA*	*γ-tubulin*
pED106	Aug2 (AN6003)-Cterm-mCherry	*pyroA*	*aug2*
pED107	Aug3 (AN5826)-Cterm-mCherry	*pyroA*	*aug3*
pED110	Aug4 (AN10254)-mCherry	*pyroA*	*pyroA*
pED114	AN0286-mCherry	*pyroA*	*pyroA*
pED120	Aug3 (AN5826)-Cterm-HAhis	*pyroA*	*aug3*
pED122	mCherry-tubulin	*pyroA*	*pyroA*
PCR fragment	Aug1 (AN3724)-Cterm-mCherry	*pyroA*	*aug1*
	*aug3Δ* construct	*pyroA*	*aug3*
	*AN0286Δ* construct	*pyroA*	*AN0286*
	*mad2Δ* construct	*pyroA*	*mad2*
	*apsBΔ* construct	*pyrG*	*apsB*

**Table 2:** PCR primers used in this study.

Name	Specificity	Sequences
*aug6* deletion	5′UTR	TTTGCGGCCGCCCCTAACAAAGGAATTG (NotI)
	5′UTR	AAAGGATCCGCTTGCATTTCTATCAGAT (BamHI)
	3′UTR	TTTATCGATGATGGTGATATTGATTGTCTG (ClaI)
	3′UTR	AAAGGGCCCTTCAGGGTCTTTACTCC (ApaI)
GFP-tagging to Aug6's C-terminus		TTTGAATTCGCATTACACCACGCACTCTC (EcoRI)
		AAATCTAGACCATCGCCCCCGGGAACGCAA (XbaI)
mCherry-tagging to γ-tubulin's C-terminus	γ-tubulin*	AAAGGTACCGTTTAAACACGCGTTGATTTATCACCTTG (KpnI/PmeI)
	γ-tubulin*	AAAGCGGCCGCATACTCCAACTTCATCCTTTC (NotI)
	mCherry	AAAGCGGCCGCATGGTGAGCAAGGGCGAGG (NotI)
	mCherry	AAAAAGCTTACTTGTACAGCTCGTCC (HindIII)
mCherry-tagging to Aug2's C-terminus		AAAGTTTAAACCCAGGTAACCCGCAATGAGC (PmeI)
		TTTGCGGCCGCAATCCGCGACTCCCACATTAC (NotI)
mCherry-tagging to Aug3's C-terminus		AAAGTTTAAACCAGAAATCTGCCACAGAACC (PmeI)
		TTTGCGGCCGCACCCCCACCTTTCCAGGAAC (NotI)
mCherry-tagging to Aug4's C-terminus		AAAGTTTAAACGATGAAGACGAGGAAGGGAG (PmeI)
		TTTGCGGCCGCACCTTTGTAATCGCCGAATCTC (NotI)
mCherry-tagging to AN0286's C-terminus		AAAGTTTAAACCTCCTCCCTTCCTCTGCATCG (PmeI)
		TTTGCGGCCGCATGATTTGAGCGCGGGTCG (NotI)
HAhis-tagging to Aug3's C-terminus	HAhis	AAAGCGGCCGCATGGTCTTTTACCCATAC (NotI)
	HAhis	AAAAAGCTTCGAGCTAGTGATGGT (HindIII)
	Aug3	AAAGTTTAAACCAGAAATCTGCCACAGAACC (PmeI)
	Aug3	TTTGCGGCCGCACCCCCACCTTTCCAGGAAC (NotI)
mCherry-tubA	tubA promoter	AAAGGTACCCGTCCGGAATATGCCACTTG (KpnI)
	tubA promoter	AAAGTTTAAACCTTGTCTAGGTGGGTGGTGA (PmeI)
	mCherry	CCCGTTTAAACACCATGGTGAGCAAGGGCGAGGAG (PmeI)
	mCherry	TTTGCGGCCGCCTTGTACAGCTCGTCCATGC (NotI)
	tubA	TTTGCGGCCGCCACCAGAGAAGTCATTAGTTTG (NotI)
	tubA	CACCGGCGCGCCTTAGTACTCAACTTCCTCACC (AscI)
mCherry-tagging to Aug1's C-terminus	Aug1	AAAGTTTAAACCAGACGAAGAGGCAGCGATG (PmeI)
	Aug1	TTTGCGGCCGCAACGTCTGTCCGACAAGTTC (NotI)
	3′UTR	GCCAAAAACCCGTATACTCCTGGA GGATGATCTACTCTTTCCCTAC (*pyroA*)
	3′UTR	GAACCGTCACATTGAGCTCG
*aug3* deletion	5′UTR	CTAGCCGCTTCGCTAATGCA
	5′UTR	GCTTTCGCGCACTTCTGCAG GTCTTGGCGCCAGCGTTGAA (*pyroA*)
	3′UTR	GCCAAAAACCCGTATACTCCTGGA TGTAAGAATTGGTGCCAAACTGG (*pyroA*)
	3′UTR	CCCACCTGGCTTTTGCAAGA
*AN0286* deletion	5′UTR	CATTGGGTGAGCCGAGTTCT
	5′UTR	GCTTTCGCGCACTTCTGCAG GTTGAGATCACAGCTTTAGCTGC (*pyroA*)
	3′UTR	GCCAAAAACCCGTATACTCCTGGA GAGAGACAGTCCAACCAACCAAT (*pyroA*)
	3′UTR	CGATCCCCAATAGTCCAGCA
*mad2* deletion	5′UTR	GACATACGCGGGAATCTTCT
	5′UTR	GCTTTCGCGCACTTCTGCAG TTTGTTCGTATATGCTGGATGAGA (*pyroA*)
	3′UTR	AACCCGTATACTCCTGGATCC ATGTGGCATGGTATCAAGACA (*pyroA*)
	3′UTR	AGATGAACAGGCAATCTACGG
*apsB* deletion	5′UTR	AGGGTGCACATCAGTGACTG
	5′UTR	CTTTCGACAGGTATCGAATTC GGTTACGGAGCAGAAAGCGTC (*pyrG*)
	3′UTR	AATCTGGTAGACAAGCACTGA TCCTCTTTCTTGCTACATATTCG (*pyrG*)
	3′UTR	CCAGCAAGCGCTTGAAACAT

The underlines indicate the restriction enzyme sites introduced for ligation.

*After cloning the γ-tubulin-mCherry fusion gene into the *pyroA* vector, the N-terminal region of γ-tubulin was deleted by PmeI/SmaI digestion. The resultant plasmid was linearised by HpaI and transformed.

### Strains

The *A. nidulans* strains used in this study are listed in Table 3. These stable strains were obtained by transformation with a linearised plasmid or direct transformation with a ‘fusion’ PCR product (in which the 5′-UTR, a selection marker, and the 3′-UTR were fused), followed by selection using an arginine, uridine, or pyridoxine marker. Transformations were performed using a procedure described previously [26]. Protoplasts were obtained by treating cells with 3 mg/mL Yatalase (Takara), and 0.3 mg/mL Lysing Enzyme (Sigma).

**Table 3:** *A. nidulans* strains used in this study.

Strain	Genotype
*Control Strain 1* *	*biA1; pyrG89; wA3; argB2; pyroA4; ligDΔ::ptrA*
*Aug6-GFP*	*biA1; pyrG89; wA3; Aug6-GFP-argB; argB2; pyroA4; ligDΔ::ptrA*
*Aug6-GFP/γ-tubulin-mCherry*	*biA1; pyrG89; wA3; Aug6-GFP-argB; argB2; γ-tubulin-mCherry-pyroA; pyroA4; ligDΔ::ptrA*
*Aug6-GFP/Aug1-mCherry*	*biA1; pyrG89; wA3; Aug6-GFP-argB; argB2; Aug1-mCherry-pyroA; pyroA4; ligDΔ::ptrA*
*Aug6-GFP/Aug2-mCherry*	*biA1; pyrG89; wA3; Aug6-GFP-argB; argB2; Aug2-mCherry-pyroA; pyroA4; ligDΔ::ptrA*
*Aug6-GFP/Aug3-mCherry*	*biA1; pyrG89; wA3; Aug6-GFP-argB; argB2; Aug3-mCherry-pyroA; pyroA4; ligDΔ::ptrA*
*Aug6-GFP/Aug4-mCherry*	*biA1; pyrG89; wA3; Aug6-GFP-argB; argB2; pyroA4::Aug4-mCherry-pyroA; ligDΔ::ptrA*
*Aug6-GFP/AN0286-mCherry*	*biA1; pyrG89; wA3; Aug6-GFP-argB; argB2; pyroA4::AN0286-mCherry-pyroA; ligDΔ::ptrA*
*Aug3-HA*	*biA1; pyrG89; wA3; argB2::argB; Aug3-HA-pyroA; pyroA4; ligDΔ::ptrA*
*Aug6-GFP/Aug3-HA*	*biA1; pyrG89; wA3; Aug6-GFP-argB; argB2; Aug3-HA-pyroA; pyroA4; ligDΔ::ptrA*
*Control Strain 2 (argB)*	*biA1; pyrG89; wA3; argB2::argB; pyroA4; ligDΔ::ptrA*
*aug6Δ*	*biA1; pyrG89; wA3; aug6Δ::argB; argB2; pyroA4; ligDΔ::ptrA*
*mCherry-tubulin*	*biA1; pyrG89; wA3; argB2::argB; pyroA4::tubA(p)-mCherry-tubA-pyroA; ligDΔ::ptrA*
*aug6Δ/mCherry-tubulin*	*biA1; pyrG89; wA3; aug6Δ::argB; argB2; pyroA4::tubA(p)-mCherry-tubA-pyroA; ligDΔ::ptrA*
*Control Strain 3 (argB, pyroA)*	*biA1; pyrG89; wA3; argB2::argB; pyroA4::pyroA; ligDΔ::ptrA*
*Control Strain 4 (argB, pyrG)*	*biA1; pyrG89::pyrG; wA3; argB2::argB; pyroA4; ligDΔ::ptrA*
*aug3Δ*	*biA1; pyrG89; wA3; argB2::argB; aug3Δ::pyroA; pyroA4; ligDΔ::ptrA*
*aug6Δ/aug3Δ*	*biA1; pyrG89; wA3; aug6Δ::argB; argB2; aug3Δ::pyroA; pyroA4; ligDΔ::ptrA*
*AN0286Δ*	*biA1; pyrG89; wA3; argB2::argB; AN0286Δ::pyroA; pyroA4; ligDΔ::ptrA*
*aug6Δ/AN0286Δ*	*biA1; pyrG89; wA3; aug6Δ::argB; argB2; AN0286Δ::pyroA; pyroA4; ligDΔ::ptrA*
*apsBΔ*	*biA1; pyrG89; wA3; argB2::argB; apsBΔ::pyrG; pyroA4; ligDΔ::ptrA*
*aug6Δ/apsBΔ*	*biA1; pyrG89; wA3; aug6Δ::argB; argB2; apsBΔ::pyrG; pyroA4; ligDΔ::ptrA*
*mad2Δ*	*biA1; pyrG89; wA3; argB2::argB; mad2Δ::pyroA; pyroA4; ligDΔ::ptrA*
*aug6Δ/mad2Δ*	*biA1; pyrG89; wA3; aug6Δ::argB; argB2; mad2Δ::pyroA; pyroA4; ligDΔ::ptrA*

*Reference: [29]. Other strains were made in this study.

### Cell culture

Using a modified version of a procedure described previously [20], *A. nidulans* germlings and hyphae were grown in complete medium (20 g/L malt extract, 2 g/L bacto tryptone, 40 g/L glucose, 1.5 mL trace elements [40 mg/L Na_2_B_4_O_7_·10H_2_O, 400 mg/L CuSO_4_·5H_2_O, 1.6 g/L FeSO4·7H_2_O, 800 mg/L MnSO_4_·4H_2_O, 800 mg/L Na_2_MoO_4_·2H_2_O, 8.0 g/L ZnSO_4_·7H_2_O], 0.25 g/L MgSO_4_, 1 g/L uridine, 0.6 g/L arginine, 10 mg/L biotin, 2.5 mg/L pyridoxine ViB6, pH = 6.5 [adjusted with HCl]) or synthetic minimal medium (6 g/L NaNO_3_, 0.52 g/L KCl, 1.52 g/L KH_2_PO_4_, 10 g/L glucose, 1.5 mL trace elements, 0.25 g/L MgSO_4_, pH = 6.5 [adjusted with NaOH]), supplemented with 1 g/L uridine, 0.525 g/L arginine, 10 mg/L biotin, and/or 2.5 mg/L pyridoxine ViB6. Conidia (asexual spores) were collected and stored in a solution containing 0.09% NaCl and 0.01% Tween-80.

### Genotyping by PCR and Southern blot analysis

Genomic DNA for PCR or Southern hybridisation was prepared using NucleoSpin Plant Midi kits (Macherey Nagel). Southern blot analysis was performed using the AlkPhos Direct kits (GE Healthcare). Both procedures were performed according to the manufacturer's instructions. In some cases, PCR genotyping was performed using the ‘colony PCR’ method, in which conidia were picked out with a toothpick, suspended in TE buffer, and added to the reaction mixture.

### Immunoprecipitation

For large-scale immunoprecipitation for mass spectrometry, conidia were inoculated into 500 mL complete medium (3×10^6^ cells/mL) and cultured at 37°C for 12 h (with shaking at 150 rpm). Cells were washed with 0.8 M NaCl solution and flash frozen in liquid nitrogen, followed by extract preparation by grinding in the presence of the lysis buffer (50 mM Tris-Cl [pH = 8.0], 150 mM NaCl, 1 mM EDTA, 0.05% Triton-X100, 1 mM DTT, and protease inhibitors). The extracts were centrifuged (9,400×*g*, 20 min), and the supernatant was incubated for 90 min with 50 µL agarose beads conjugated with anti-GFP (or HA) antibody (MBL, Japan or Sigma, respectively). The beads were washed 3 times with the extraction buffer, followed by elution of the precipitated proteins with urea solution (10 mM Tris-Cl, 8 M urea, 100 mM NaH_2_PO_4,_ pH = 4.5). The eluate was precipitated with TCA, followed by boiling in the SDS sample buffer. In one case, we omitted the elution process and added the SDS sample buffer directly to the beads. For smaller-scale immunoprecipitation, conidia were inoculated into 50 mL complete medium (3×10^6^ cells/mL) and cultured at 37°C for 12 h. Mitotic cells (prometaphase or metaphase) were accumulated by treating cells with 1.2 µg/mL benomyl for 60 min, followed by drug washout and culturing for 2.5 min. Cells were then flash frozen with liquid nitrogen.

### Mass spectrometry

Immunoprecipitants were separated on a 10–20% gradient gel and size-fractionated by horizontally slicing the gel into 16 pieces. Each piece of the gel was digested with trypsin for the LC/MS/MS analysis, as described previously [27]. The digested peptides were analysed using a capillary liquid chromatography system (Ultima3000; DIONEX) connected online to a mass spectrometer (LTQ-XL, Thermo Scientific) [28]. Raw spectra data were processed using the software SEQUEST to extract peak lists. The obtained peak lists were analysed using the MASCOT program against an *A. nidulans* protein database extracted from the Aspergillus Genome Database (AspGD: http://www.aspergillusgenome.org/).

### Microscopy

Time-lapse imaging of the hyphae was performed using 2 methods. For the first method, 3×10^5^ conidia were inoculated into 300 µL minimal medium in 8-well, glass-bottom chambers (IWAKI), and incubated for 7 h at 37°C or 18 h at room temperature. For the second, more recently developed method, we inoculated 3×10^5^ conidia onto 1 mL of agar medium in 3.5-cm cell culture dishes and incubated them for 7 h at 37°C. We then transferred the agar pad upside down onto 6-well, glass-bottom plates (IWAKI) on which 40 µL minimal media had been spotted. Imaging was performed at 23–25°C using a spinning-disc confocal microscope equipped with an EMCCD camera (CSU-X1 [Yokogawa], TE2000 [Nikon], and ImagEM [Hamamatsu], 100× objective lens [1.40 NA]; these devices were controlled using the software Micro-manager). Immunostaining was performed for hyphae grown on the 22×22 mm coverslips placed in the 3.5-cm cell culture dishes. Prior to fixation, 3×10^6^ conidia were inoculated into 2 mL complete medium and cultured for 7 h at 37°C or 18 h at room temperature. Cells were fixed for 30 min at 37°C using 1.5 mL of 3.2% paraformaldehyde, 50 mM PIPES-KOH (pH = 6.8), 5 mM MgSO_4,_ 5% DMSO, and 25 mM EGTA. The fixed cells were washed twice with PE Buffer (50 mM PIPES-KOH [pH = 6.8], 25 mM EGTA), followed by cell wall digestion (1 h at room temperature with 1.5 mL of 50 mM sodium citrate [pH = 5.8], 1 mM MgSO_4_, 2.5 mM EGTA, 2% BSA, 10 mg/mL driserase, 1 mg/mL lyticase, and 16 mg/mL lysing enzyme). Anti-α-tubulin (YOL 1/34; 1∶1000 [Rat]) and anti-γ-tubulin (GTU88; 1∶1000 [Mouse]) were used for staining, and the samples were imaged using a spinning-disc confocal microscope.

### Gel filtration chromatography

For gel filtration chromatography, whole-cell extracts of the Aug6-GFP strain (cultured in 50 mL for 12 h) was centrifuged for 20 min at 106,000×*g*, followed by glass-fibre filtration (Millex-AP 0.20 µm). The cleared extract (1 mL) was fractionated using a Superdex 200 10/300 column attached to a Biorad BioLogicDuoFlow system, followed by immunoblotting using anti-GFP (JL-8; 1∶1000 [Mouse]). The size marker used in the chromatography analysis was described in [8].

### MT cosedimentation assay

MT was formed by incubating 260 µg pig tubulins with 2 mM GTP, 1 mM DTT, 20 µM taxol in 200 µL of MRB80 buffer (80 mM PIPES-KOH [pH = 6.8], 4 mM MgCl_2_, 1 mM EGTA) (37°C, 20 min). Unpolymerised tubulins were removed by centrifugation at 106,000×*g* for 10 min at 30°C, and the MT pellet was resuspended with 80 µL MRB80 containing 20 µM taxol. *A. nidulans* extracts in 900 µL lysis buffer (80 mM PIPES-KOH [pH = 6.8], 1 mM EDTA, 0.05% Triton-X100, 1 mM DTT, and protease inhibitors) were prepared from the 30-mL cultures. Protein aggregates were removed by centrifugation at 208,000×*g* for 20 min at 4°C. Taxol-stabilised MTs (20 µL) and the cell extracts (90 µL) were mixed for 15 min at room temperature, followed by centrifugation at 106,000×*g* for 10 min at 25°C. The supernatant and pellet were immunoblotted with the anti-GFP antibody (JL-8; 1∶1000).

## Supporting Information

Movie S1 **Time-lapse imaging of Aug6-GFP and γ-tubulin-mCherry during mitosis.** A cell expressing Aug6-GFP and γ-tubulin-mCherry was imaged every 20 s using a spinning-disc confocal microscope (24°C in the minimal medium). Bar, 2 µm.
(MOV)

Movie S2 **Time-lapse imaging of Aug6-GFP and mCherry-tagged Aug1, Aug2, Aug3, Aug4, or AN0286.** Cells were imaged every 15–30 s using a spinning-disc confocal microscope (24°C in the minimal medium). Location of the mitotic spindle is indicated by arrows. Granule signals are autofluorescence, which were detected regardless of the expression of GFP/mCherry. Bar, 2 µm.
(MOV)

Movie S3 **Time-lapse imaging of mCherry-tubulin in WT and** ***aug6Δ*** **.** Control and *aug6Δ* cells expressing mCherry-tubulin were imaged every 20 s using a spinning-disc confocal microscope (24°C in the minimal medium). Displayed are the maximum projections of 5 z-section images (every 0.25 µm). Bar, 2 µm.
(MOV)
